# Effect of neoadjuvant radiotherapy on survival of non-metastatic pancreatic ductal adenocarcinoma: a SEER database analysis

**DOI:** 10.1186/s13014-020-01561-z

**Published:** 2020-05-13

**Authors:** Dan Wang, Chongshun Liu, Yuan Zhou, Tingyu Yan, Chenglong Li, Qionghui Yang, Yang Xu, Lilan Zhao, Qian Pei, Fengbo Tan, Cenap Güngör, Yuqiang Li

**Affiliations:** 1 Department of General Visceral and Thoracic Surgery, University Medical Center Hamburg-Eppendorf, Hamburg, Germany; 2 Department of Gastrointestinal Surgery, Xiangya Hospital, Central South University, Changsha, China; 3 George Washington University Hospital, Washington, USA; 4 Department of Ophthalmology, The Fourth Affiliated Hospital of China Medical University, Shenyang, China; 5 Department of Pediatrics, Yueqing Third People’s Hospital, Yueqing, China; 6 Department of Thoracic Surgery, Fujian Provincial Hospital, Fuzhou, China

**Keywords:** Neoadjuvant radiotherapy, Pancreatic ductal adenocarcinoma, Overall survival, SEER, Propensity score matching

## Abstract

**Background:**

Neoadjuvant radiotherapy has been shown to improve marginal negative resection and local control of Pancreatic Ductal Adenocarcinoma (PDAC). However, whether it improves overall survival (OS) in patients with non-metastatic PDAC remains controversial. Therefore, the purpose of this study was to analyze the benefits of only surgery, neoadjuvant radiotherapy, adjuvant radiotherapy, and surgery plus chemotherapy for OS in patients with non-metastatic PDAC.

**Methods:**

PDAC diagnosed by surgical histopathology in the Surveillance, Epidemiology, and End Results (SEER) database between 2004 and 2016 was selected. Kaplan-Meier analysis was used to compare the prognosis of patients with different treatments. Cox proportional risk model was used to analyze independent predictors of OS. Propensity score matching (PSM) was used to analyze the tumor prognosis of different treatment methods.

**Results:**

Before PSM analysis, the OS of surgery plus chemotherapy (HRs = 0.896, 95%CIs, 0.827–0.970; *P* = 0.007) were significantly better than the other three treatments for stage T1-3N0M0 PDAC patients. For stage T1-3N + M0 patients, adjuvant radiotherapy (HRs = 0.613, 95% CIs, 0.579–0.649; *P* < 0.001) had significantly better OS than surgery plus chemotherapy and neoadjuvant radiotherapy. For stage T4N0M0 patients, neoadjuvant radiotherapy (HRs = 0.482, 95% CIs, 0.347–0.670; *P* < 0.001) had significantly better OS than surgery plus chemotherapy and adjuvant radiotherapy. For stage T4N + M0 patients, neoadjuvant radiotherapy (HRs = 0.338, 95% CIs, 0.215–0.532; *P* < 0.001) had significantly longer OS than adjuvant radiotherapy and surgery plus chemotherapy. Even after PSM, Chemotherapy plus surgery was still the best treatment for T1-3N0M0 patients. Postoperative adjuvant radiotherapy had the best prognosis among T1-3N + M0 patients, and neoadjuvant radiotherapy was the best treatment for T4 patients.

**Conclusions:**

For patients with non-metastatic PDAC, neoadjuvant radiotherapy, adjuvant radiotherapy and surgery plus chemotherapy were superior to only surgery in OS. For patients with stage T4 non-metastatic PDAC, neoadjuvant radiotherapy had the potential to be strongly recommended over adjuvant radiotherapy and surgery plus chemotherapy. However, neoadjuvant radiotherapy failed to benefit the survival of T1-3N0M0 stage patients, and surgery plus chemotherapy was preferred. For T1-3N + M0, neoadjuvant radiotherapy had no obvious advantage over adjuvant radiotherapy or surgery plus chemotherapy in OS, and adjuvant radiotherapy was more recommended.

## Background

Pancreatic Ductal Adenocarcinoma (PDAC) is a highly malignant tumor with a 5-year survival of about 7% and is on pace to become the second leading cause of cancer-related death in the United States by 2030 [1, 2].The main reasons for this frustrating survival are the lack of specific diagnostic methods in early PDAC, the high aggressiveness of the tumor, and the early metastasis [3, 4]. More than 80% of PDAC patients already have locally advanced unresectable or metastatic disease at the time of diagnosis [5]. Moreover, only about 20% of PDAC patients who underwent surgical resection achieve long-term remission, which may be related to the high rate of recurrence after surgery [6].

Neoadjuvant therapy is gaining more and more attention from physicians and scholars due to the dismal survival. Based on a retrospective analysis of significant adjuvant chemotherapy studies in the 1970s, Frei firstly proposed the concept of neoadjuvant therapy (chemotherapy before surgery), which extended disease-free survival in 1982 [7]. Then, the further study of neoadjuvant therapy included preoperative radiotherapy and chemoradiotherapy. In 1990, the term of neoadjuvant therapy was first used in PDAC. Fox Chase cancer center reported that neoadjuvant chemotherapy and radiotherapy improved the resectability of locally advanced PDAC [8]. Moreover, the treatment model for PDAC was changed from “surgical-first” to “multi-disciplinary team” (MDT) with advances in medical technology and treatment concepts in the past decades [9]. It is widely recognized regarding the application of neoadjuvant chemotherapy for patients with PDAC today [10, 11]. However, the role of neoadjuvant radiotherapy in PDAC is still under debate due to the lack of relatively reliable data. Currently, neoadjuvant radiotherapy is mainly used for borderline resectable PDAC and locally advanced PDAC since which may improve the marginal negative resection rate and local control rate [12, 13]. However, it is unclear whether neoadjuvant radiotherapy improves survival of patients with PDAC. In addition, it is still highly controversial and requires further discussion about whether patients with initially resectable PDAC can get benefit from neoadjuvant radiotherapy.

The Surveillance, Epidemiology, and End Results (SEER) Program collects data on cancer diagnosis, treatment, and survival for approximately 30% of the U.S. population. We attempted to use the SEER database to analyze the effects of different treatment methods including surgery-limited, neoadjuvant radiotherapy, adjuvant radiotherapy and surgery plus chemotherapy on overall survival (OS) in patients with non-metastatic PDAC.

## Materials and methods

### Data source

**Fig. 1:**
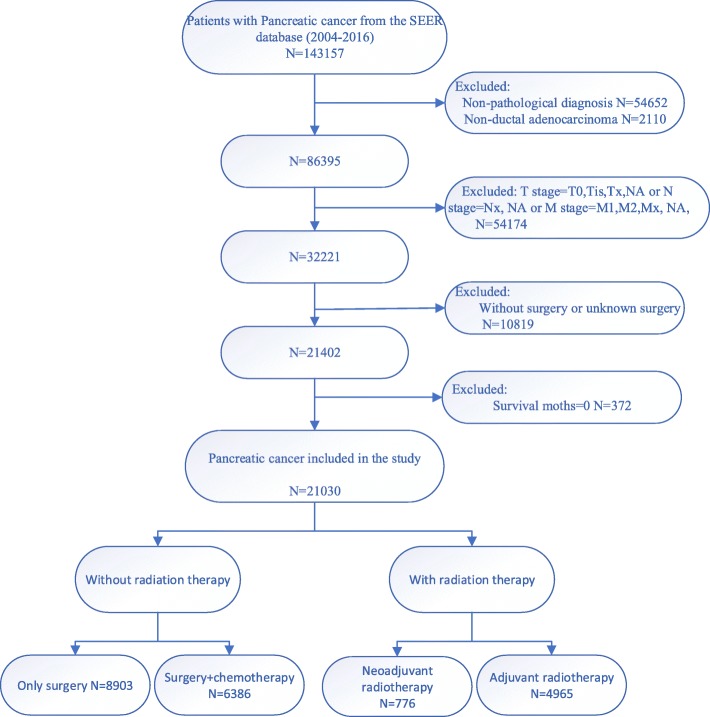
Procedures for inclusion and exclusion of PDAC patients

The cohort used in this study was created from custom data (additional treatment fields) from SEER 18 Registries, and a report was submitted in November 2018 (varied from 1973 to 2016). PDAC diagnosed by surgical histopathology between 2004 and 2016 was selected. In addition, we included basic patient information, detailed clinical staging data, as well as follow-up information, tumor size, and treatment options. Combined with tumor size, T and N staging were recorded on the basis of the 8th edition of TNM staging system. The study was limited to patients with non-metastatic PDAC (any T with any N and M0). After excluding patients who had not undergone surgery and classifying radiotherapy as “no radiation”, “radiation after surgery”, “radiation prior to surgery” and “no/unknown”, 21,030 patients were contained in the study (Fig. 1). The patients were divided into the following four groups according to the treatment methods: 1. Only surgery group (No radiation or chemotherapy); 2. Surgery + chemotherapy group (without radiation); 3. Neoadjuvant radiotherapy group (Neoadjuvant radiotherapy + surgery with or without chemotherapy); 4. Adjuvant radiotherapy group (surgery + adjuvant radiotherapy with or without chemotherapy). The International Study Group on Pancreatic Surgery (ISGPS) consensus recommended that the number of lymph nodes examined should be at least > 15. Therefore, the number of regional nodes examined was divided into three groups: < 15, ≥15 and unknown.

### Statistical analyses

Chi-square test was utilized to compare the classification data. Kaplan-Meier method was used to estimate the survival probability and log-rank test was applied to evaluate the significance difference of OS. Only variables significantly associated with survival in univariate Cox analysis were contained in multivariate Cox analysis. Cox proportional risk model was used to analyze the relationship between patients’ clinical characteristics and treatment methods and their survival. Univariate and multivariate models were used to assess the Hazard ratios (HRs) and 95% confidence interval (CIs). The oncological outcomes of different treatments were analyzed by propensity score matching (PSM) analysis. SPSS 25.0 (IBM, Armonk, NY, USA) was used for statistical analysis in this study, and all *p* values less than 0.05 were statistically significant.

## Results

### Basic characteristics of the patients

The basic demographic characteristics of all the patients in this study were shown in Table 1. The majority of the patients were married and Caucasians. 55.17% of the target population were over 65 years old and about 50.68% were males. Most of the patients (64.56%) had tumor lesions in the head of the pancreas. Moderately differentiated tumors (41.26%) constituted the majority of the population. Among the 21,030 patients, most of them were stage T2, accounting for 50.87% (10,699 patients), and about 4.60% (968 patients) were stage T4. In terms of the treatment regimen, about 42.33% of patients only were managed with surgical treatment, about 30.37% received surgery plus chemotherapy, about 23.61% received postoperative adjuvant radiotherapy, and only about 3.69% underwent neoadjuvant radiotherapy.

**Table 1:** The basic and clinical features of non-metastatic PDAC

Characteristics	Level	Number (%)
Insurance Recode	Insured	17,218(81.87%)
	No/unknown	3812(18.13%)
Marital status	Married	13,291(63.20%)
	Single	6984(33.21%)
	Unknown	755(3.59%)
Age, years	< 65	9427(44.83%)
	≥65	11,603(55.17%)
Race recode	White	17,170(81.65%)
	Other	3860(18.35%)
Sex	Male	10,657(50.68%)
	Female	10,373(49.32%)
Tumor site	Pancreas Head	13,576(64.56%)
	Pancreas Body Tail	5175(24.61%)
	Pancreas Other	2279(10.83%)
Grade	I	4147(19.72%)
	II	8677(41.26%)
	III/IV	6075(28.89%)
	Unknown	2131(10.13%)
T stage	T1	4242(20.17%)
	T2	10,699(50.87%)
	T3	5121(24.36%)
	T4	968(4.60%)
N stage	N0	9467(45.02%)
	N1	7400(35.19%)
	N2	4163(19.79%)
Treatment methods	Only surgery	8903(42.33%)
	Surgery + chemotherapy	6386(30.37%)
	Neoadjuvant radiotherapy	776(3.69%)
	Adjuvant radiotherapy	4965(23.61%)
Regional nodes examined	< 15	11,410(54.26%)
	≥15	9437(44.87%)
	Unknown	183(0.87%)

*PDAC* Pancreatic Ductal Adenocarcinoma

### Survival analysis before propensity score matching

**Fig. 2:**
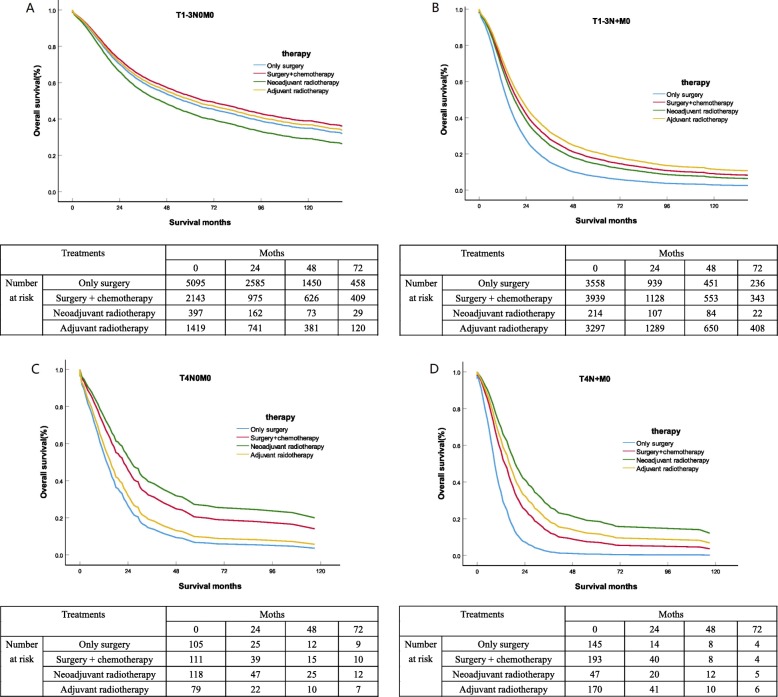
OS estimated with the Kaplan-Meier method for non- metastatic PDAC. A.OS estimated with the Kaplan-Meier method for PDAC patients with T1-3N0M0 stage receiving different treatment methods (surgery alone versus (vs.) adjuvant radiotherapy: *p* = 0.223; surgery alone vs. neoadjuvant radiotherapy: *p* = 0.027; surgery alone vs. surgery plus chemotherapy: *p* = 0.007;adjuvant radiotherapy vs. surgery plus chemotherapy: *p* = 0.023). B. OS estimated with the Kaplan-Meier method for PDAC patients with T1-3N + M0 stage receiving different treatment methods (surgery alone versus (vs.) adjuvant radiotherapy: *p* < 0.001; surgery alone vs. neoadjuvant radiotherapy: *p* = 0.001; surgery alone vs. surgery plus chemotherapy: p < 0.001;adjuvant radiotherapy vs. neoadjuvant radiotherapy: *p* = 0.017;adjuvant radiotherapy vs. surgery plus chemotherapy: *p* < 0.001). C. OS estimated with the Kaplan-Meier method for PDAC patients with T4N0M0 stage receiving different treatment methods (surgery alone versus (vs.) adjuvant radiotherapy: *p* = 0.353; surgery alone vs. neoadjuvant radiotherapy: *p* < 0.001; surgery alone vs. surgery plus chemotherapy: *p* = 0.001;adjuvant radiotherapy vs. neoadjuvant radiotherapy: *p* < 0.001;adjuvant radiotherapy vs. surgery plus chemotherapy: *p* < 0.001;neoadjuvant radiotherapy vs. surgery plus chemotherapy: *p* < 0.001). D. OS estimated with the Kaplan-Meier method for PDAC patients with T4N + M0 stage receiving different treatment methods (surgery alone versus (vs.) adjuvant radiotherapy: *p* = 0.353; surgery alone vs. neoadjuvant radiotherapy: *p* < 0.001; surgery alone vs. surgery plus chemotherapy: *p* < 0.001;adjuvant radiotherapy vs. neoadjuvant radiotherapy: *p* < 0.001;adjuvant radiotherapy vs. surgery plus chemotherapy: p < 0.001;neoadjuvant radiotherapy vs. surgery plus chemotherapy: *p* < 0.001)

Using univariate and multivariate Cox proportional risk analysis for the total population (Table 2), uninsured status, single status, advanced age (≥65 years old), pancreatic head tumor, high tumor grade, tumor T, N stage, therapy methods and regional nodes examined < 15 were all relevant to poor prognosis (all *P* < 0.001). The Kaplan Meier curve of overall survival in PDAC patients were shown in Fig. 2. For patients with non-metastatic PDAC, neoadjuvant radiotherapy, adjuvant radiotherapy and surgery plus chemotherapy had significantly better OS than surgery alone (*P* < 0.001). After adjusting for insurance status, marital status, age, race, gender, tumor site, tumor grade, T stage, N stage and regional nodes examined, multivariate Cox analysis of different treatment methods was performed, and the influence of each group on OS was shown in Table 3. The mean 1-, 3-year survival rates for PDAC patients were shown in Table 4.

**Table 2:** Univariate and multivariate analysis for OS of all patients(*n* = 21,030)

Characteristics	Level	Univariate analysis	Multivariate analysis		
		*P*	HR	95%CI	*P*
Insurance Recode		< 0.001			< 0.001
	Insured		Reference	Reference	Reference
	No/unknown		1.201	1.152–1.253	< 0.001
Marital status		< 0.001			< 0.001
	Married		Reference	Reference	Reference
	Single		1.140	1.098–1.184	< 0.001
	Unknown		0.985	0.891–1.088	0.760
Age, years		< 0.001			< 0.001
	< 65		Reference	Reference	Reference
	≥65		1.436	1.384–1.489	< 0.001
Race recode		< 0.001			0.508
	White		Reference	Reference	Reference
	Other		1.016	0.970–1.065	0.508
Sex		< 0.001			< 0.001
	Female		Reference	Reference	Reference
	Male		1.106	1.068–1.147	< 0.001
Tumor site		< 0.001			< 0.001
	Pancreas Head		Reference	Reference	Reference
	Pancreas Body Tail		0.704	0.670–0.739	< 0.001
	Pancreas Other		0.844	0.795–0.897	< 0.001
Grade		< 0.001			< 0.001
	I		Reference	Reference	Reference
	II		2.409	2.260–2.567	< 0.001
	III/IV		3.274	3.064–3.498	< 0.001
	Unknown		1.578	1.449–1.719	< 0.001
T stage		< 0.001			< 0.001
	T1		Reference	Reference	Reference
	T2		1.407	1.335–1.483	< 0.001
	T3		1.595	1.503–1.692	< 0.001
	T4		2.396	2.192–2.620	< 0.001
N stage		< 0.001			< 0.001
	N0		Reference	Reference	Reference
	N1		1.617	1.550–1.688	< 0.001
	N2		2.078	1.975–2.186	< 0.001
Treatment methods		< 0.001			< 0.001
	Only surgery		Reference	Reference	Reference
	Surgery + chemotherapy		0.773	0.739–0.809	< 0.001
	Neoadjuvant radiotherapy		0.855	0.776–0.943	0.002
	Adjuvant radiotherapy		0.719	0.686–0.752	< 0.001
Regional nodes examined		< 0.001			< 0.001
	< 15		Reference	Reference	Reference
	≥15		0.828	0.797–0.859	< 0.001
	Unknown		1.133	0.954–1.346	0.156

*OS* Overall Survival, *CI* Confidence intervals, *HR* Hazard ratios

**Table 3:** Multivariate Cox analyses of treatment methods for OS (*n* = 21,030)

TNM Stage	Treatments	Multivariate HR (95% CI)	*P* value
T1-3N0M0			0.001
	Only surgery	Reference	
	Surgery + chemotherapy	0.896(0.827–0.970)	0.007
	Neoadjuvant radiotherapy	1.171(1.019–1.347)	0.027
	Adjuvant radiotherapy	0.950(0.874–1.032)	0.223
T1-3N + M0			< 0.001
	Only surgery	Reference	
	Surgery + chemotherapy	0.686(0.649–0.726)	< 0.001
	Neoadjuvant radiotherapy	0.751(0.635–0.887)	0.001
	Adjuvant radiotherapy	0.613(0.579–0.649)	< 0.001
T4N0M0			< 0.001
	Only surgery	Reference	
	Surgery + chemotherapy	0.588(0.424–0.814)	0.001
	Neoadjuvant radiotherapy	0.482(0.347–0.670)	< 0.001
	Adjuvant radiotherapy	0.858(0.621–1.185)	0.353
T4N + M0			< 0.001
	Only surgery	Reference	
	Surgery + chemotherapy	0.530(0.411–0.683)	< 0.001
	Neoadjuvant radiotherapy	0.338(0.215–0.532)	< 0.001
	Adjuvant radiotherapy	0.430(0.334–0.554)	< 0.001

*OS* Overall Survival, *CI* Confidence intervals, *HR* Hazard ratios

**Table 4:** Median survival and, 1-, 3-year OS of PDAC patients (*n* = 21,030)

TNM Stage	Treatments	Median survival	1-year OS	3-year OS
T1-3N0M0	Only surgery	21	70.14%	46.52%
	Surgery + chemotherapy	25	73.27%	49.36%
	Neoadjuvant radiotherapy	19	67.65%	39.87%
	Adjuvant radiotherapy	24	72.09%	48.16%
T1-3N + M0	Only surgery	10	38.65%	6.88%
	Surgery + chemotherapy	15	42.27%	14.85%
	Neoadjuvant radiotherapy	16	39.26%	12.34%
	Adjuvant radiotherapy	19	47.64%	18.63%
T4N0M0	Only surgery	7	27.14%	6.72%
	Surgery + chemotherapy	17	47.67%	19.36%
	Neoadjuvant radiotherapy	20	52.75%	26.47%
	Adjuvant radiotherapy	14	31.82%	8.73%
T4N + M0	Only surgery	6	7.28%	0.05%
	Surgery + chemotherapy	10	26.18%	6.54%
	Neoadjuvant radiotherapy	17	42.34%	16.56%
	Adjuvant radiotherapy	16	33.29%	9.86%

*PDAC* Pancreatic Ductal Adenocarcinoma, *OS* Overall Survival

The OS of surgery plus chemotherapy (HRs =0.896, 95%CIs, 0.827–0.970; *P* = 0.007) were significantly better than the other three treatments in stage T1-3N0M0 PDAC patients. Adjuvant radiotherapy (HRs = 0.950; 95% CIs, 0.874–1.032; *P* = 0.223), and only surgery had similar OS results. However, neoadjuvant radiotherapy (HRs = 1.171;95% CIs, 1.019–1.347; *P* = 0.027) seems to be a risk factor for OS. The median survival for only surgery, surgery plus chemotherapy, neoadjuvant radiotherapy and adjuvant radiotherapy was 21months, 25months, 19months, 24months, respectively. Adjuvant radiotherapy (HRs = 0.613, 95% CIs, 0.579–0.649; *P* < 0.001) had significantly better OS results than surgery plus chemotherapy (HRs = 0.686; 95% CIs, 0.649–0.726;*P* < 0.001) and neoadjuvant radiotherapy (HRs = 0.751; 95% CIs, 0.635–0.887; *P* = 0.001) in stage T1-3N + M0 patients, with median survival of 19 months, 15 months, and 16 months, respectively. Specially, for stage T4N0M0 patients, neoadjuvant radiotherapy (HRs = 0.482, 95% CIs, 0.347–0.670; *P* < 0.001) had significantly better OS outcomes than surgery plus chemotherapy (HRs = 0.588; 95% CIs, 0.424–0.814; P = 0.001) and adjuvant radiotherapy (HRs = 0.858; 95% CIs, 0.621–1.185; *P* = 0.353), with median survival of 20 months, 17 months, and 14 months, respectively. Similarly, for stage T4N + M0 patients, neoadjuvant radiotherapy (HRs = 0.338, 95% CIs, 0.215–0.532; *P* < 0.001) had significantly longer OS outcomes than adjuvant radiotherapy (HRs = 0.430; 95% CIs, 0.334–0.554; *P* < 0.001) and surgery plus chemotherapy (HRs = 0.530; 95% CIs, 0.411–0.683; *P* < 0.001), with median survival of 17 months, 16 months, and 10 months, respectively.

### Survival analysis after propensity score matching

The balanced population of the neoadjuvant radiotherapy group and the only surgery group(*n* = 296), the neoadjuvant radiotherapy group and the adjuvant radiotherapy group(*n* = 208), the neoadjuvant radiotherapy group and the surgery plus chemotherapy group (*n* = 288) were obtained by multiple 1:1 propensity score matching for stage T1-3N0M0 PDAC patients. Before and after the PSM, the results of univariate and multivariate analyses of OS in different groups were shown in Table 5. The OS of the neoadjuvant radiotherapy group was no better than that of the adjuvant radiotherapy group (HRs = 0.807; 95% CIs, 0.649–1.035; *P* = 0.125) and the only surgery group (HRs = 1.164; 95% CIs, 0.934–1.449; *P* = 0.176), while the OS of the surgery plus chemotherapy group was better than that of the neoadjuvant radiotherapy group (HRs = 1.280; 95% CIs, 1.045–1.574; *P* = 0.025) in stage T1-3N0M0 PDAC patients.

**Table 5:** Univariate and Multivariate Cox analyses of treatment methods for OS after PSM

		Before PSM				After PSM			
		Univariate analysis	Multivariate analysis			Univariate analysis	Multivariate analysis		
TNM stage	Treatment	*P*	HR	95%CI	P	P	HR	95%CI	P
T1-3N0M0		< 0.001			0.038	0.048			0.176
Only surgery			Reference	Reference	Reference		Reference	Reference	Reference
Neoadjuvant radiotherapy			1.486	1.315–1.679	0.038		1.164	0.934–1.449	0.176
T1-3N0M0		0.016			0.006	0.039			0.125
Neoadjuvant radiotherapy			Reference	Reference	Reference		Reference	Reference	Reference
Adjuvant radiotherapy			0.809	0.695–0.941	0.006		0.807	0.649–1.035	0.125
T1-3N0M0		0.001			0.001	0.034			0.025
Surgery plus chemotherapy			Reference	Reference	Reference		Reference	Reference	Reference
Neoadjuvant radiotherapy			1.285	1.107–1.491	0.001		1.280	1.045–1.574	0.025
T1-3N + M0		0.002			0.008	0.049			0.036
Only surgery			Reference	Reference	Reference		Reference	Reference	Reference
Neoadjuvant radiotherapy			0.795	0.671–0.942	0.008		0.618	0.429–0.863	0.036
T1-3N + M0		0.019			0.015	0.040			0.022
Adjuvant radiotherapy			Reference	Reference	Reference		Reference	Reference	Reference
Neoadjuvant radiotherapy			1.047	1.013–1.706	0.015		1.364	1.046–1.777	0.022
T1-3N + M0		0.035			0.795	0.021			0.541
Surgery plus chemotherapy			Reference	Reference	Reference		Reference	Reference	Reference
Neoadjuvant radiotherapy			1.023	0.863–1.212	0.795		1.083	0.838–1.400	0.541
T4NxM0		< 0.001			< 0.001	< 0.001			< 0.001
Only surgery			Reference	Reference	Reference		Reference	Reference	Reference
Neoadjuvant radiotherapy			0.459	0.349–0.602	< 0.001		0.466	0.331–0.657	< 0.001
T4NxM0		< 0.001			< 0.001	0.002			0.002
Adjuvant radiotherapy			Reference	Reference	Reference		Reference	Reference	Reference
Neoadjuvant radiotherapy			0.590	0.445–0.781	< 0.001		0.589	0.419–0.830	0.002
T4NxM0		< 0.001			0.040	0.028			0.028
Surgery plus chemotherapy			Reference	Reference	Reference		Reference	Reference	Reference
Neoadjuvant radiotherapy			0.752	0.573–0.987	0.040		0.707	0.519–0.963	0.028

*PSM* Propensity score matching, *OS* Overall Survival, *CI* Confidence intervals, *HR* Hazard ratios

Similarly, the 1:1 propensity score matching was used to obtain the balanced population of the neoadjuvant radiotherapy group and the only surgery group(*n* = 155), neoadjuvant radiotherapy group and the adjuvant radiotherapy group (*n* = 153), neoadjuvant radiotherapy group and the surgery plus chemotherapy group (*n* = 166) in stage T1-3N + M0 PDAC patients. The OS of the neoadjuvant radiotherapy group was better than that of the only surgery group (HRs = 0.618; 95% CIs, 0.429–0.863; *P* = 0.036), but there was no difference with that of the operation plus chemotherapy group (HRs = 1.083; 95% CIs, 0.838–1.400; *P* = 0.541). The adjuvant radiotherapy group had the best prognosis (HRs = 1.364; 95% CIs, 1.046–1.777; *P* = 0.022).

**Fig. 3:**
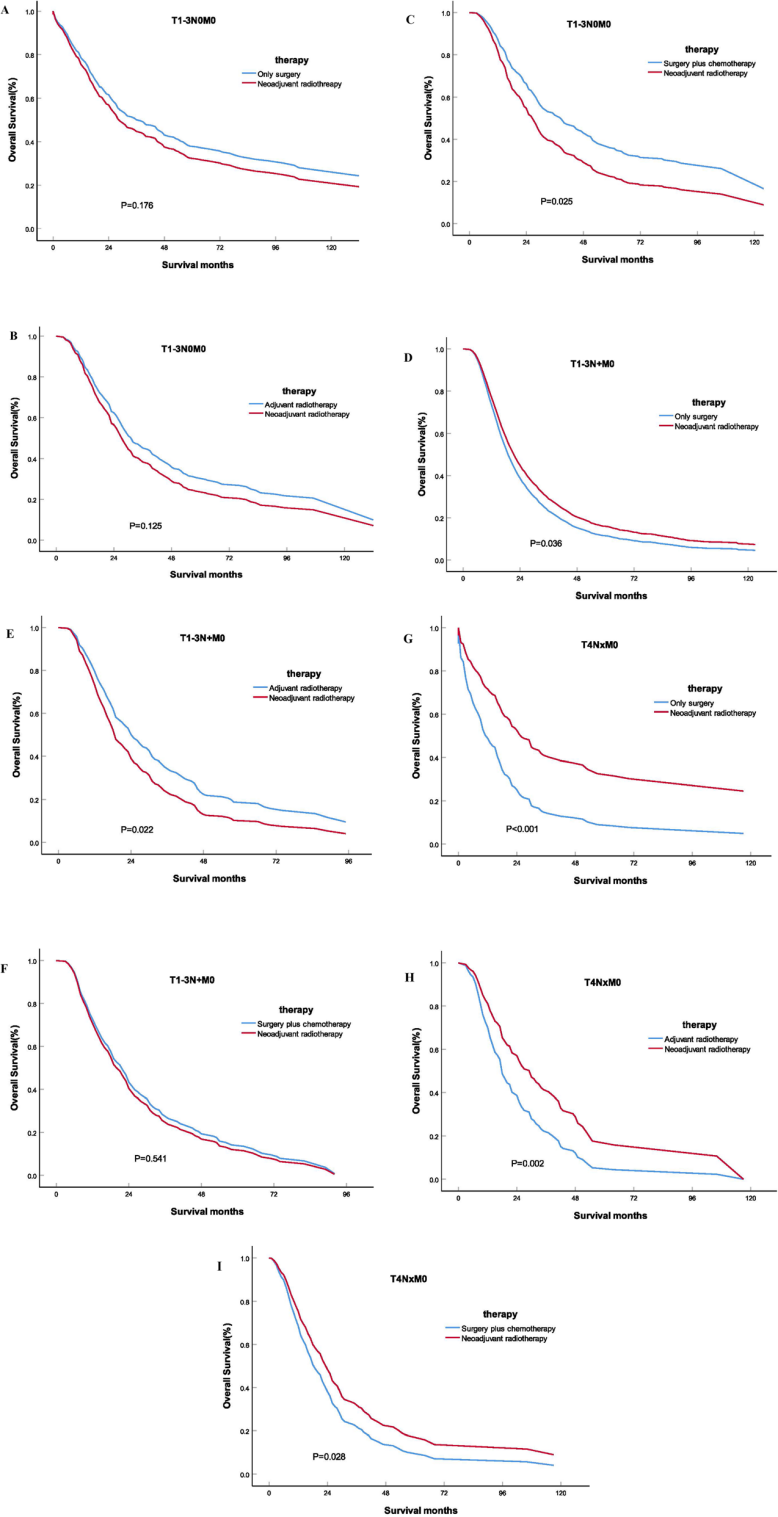
The Kaplan-Meier curve of overall survival of PDAC patients after PSM. A.Comparison of OS between the neoadjuvant radiotherapy group and the only surgery group for T1-3N0M0 stage (*P* = 0.176); B. Comparison of OS between the neoadjuvant radiotherapy group and the adjuvant radiotherapy group for T1-3N0M0 stage (*P* = 0.125); C. Comparison of OS between the neoadjuvant radiotherapy group and the surgery plus chemotherapy group for T1-3N0M0 stage (*P* = 0.025); D. Comparison of OS between the neoadjuvant radiotherapy group and the only surgery group for T1-3N + M0 stage (*P* = 0.036); E. Comparison of OS between the neoadjuvant radiotherapy group and the adjuvant radiotherapy group for T1-3N + M0 stage (*P* = 0.022); F. Comparison of OS between the neoadjuvant radiotherapy group and the surgery plus chemotherapy group for T1-3N + M0 stage (*P* = 0.541); G. Comparison of OS between the neoadjuvant radiotherapy group and the only surgery group for T4 stage (*P* < 0.001); H. Comparison of OS between the neoadjuvant radiotherapy group and the adjuvant radiotherapy group for T4 stage (*P* = 0.002); I. Comparison of OS between the neoadjuvant radiotherapy group and the surgery plus chemotherapy group for T4 stage (*P* = 0.028).

The 1:1 propensity score matching was used to obtain the balanced population of the neoadjuvant radiotherapy group and the only surgery group(*n* = 104), neoadjuvant radiotherapy group and the adjuvant radiotherapy group (*n* = 102), neoadjuvant radiotherapy group and the surgery plus chemotherapy group (*n* = 138) in stage T4 PDAC patients. The OS of neoadjuvant radiotherapy group was better than that of only surgery group (HRs = 0.466; 95% CIs, 0.331–0.657; *P* < 0.001), adjuvant radiotherapy group (HRs = 0.589; 95% CIs, 0.419–0.830; *P* = 0.002) and surgery plus chemotherapy group (HRs = 0.707; 95% CIs, 0.519–0.963; *P* = 0.028). The Kaplan-Meier curve of overall survival of PDAC patients after PSM were shown in Fig. 3.

## Discussion

The surgical approach for PDAC mainly depends on the anatomical location of the tumor. Although the surgical resection rate and surgical safety of PDAC have been significantly improved, and the incidence of serious complications during perioperative period has been significantly reduced in the past 30 years, the main goal still remains the same: removal of all lesions visible to the naked eye and microscopically within the pancreas and drainage of the lymph nodes, known as marginal negative or R0 resection [14]. However, even after R0 resection, the prognosis of PDAC is not significantly improved, and the treatment of PDAC remains extremely challenging [9]. In this study, patients with non-metastatic PDAC who received surgery alone had the worst prognosis. The main reason for this poor prognosis is local recurrence or distant metastasis of PDAC after surgery, which is a key factor affecting the long-term survival of patients. This showed that only surgical treatment of PDAC is far from enough, and we need to combine systematic adjuvant therapy. Therefore, the application value of neoadjuvant radiotherapy in PDAC has gradually become a hot topic, but there is still a big controversy.

The focus of this study was to determine whether neoadjuvant radiotherapy had a better effect on OS than postoperative radiotherapy, but the existing evidence remains controversial. Currently, it is generally accepted that neoadjuvant radiotherapy is superior to adjuvant radiotherapy mainly related to tumor response and preservation of normal tissues, including the following points [1]. The goal of neoadjuvant radiotherapy is to reduce the stage of the tumor and, in combination with R0 resection, increase the chance of survival. With effective treatment, a percentage of potentially unresectable tumors may be reduced in staging in order to be surgically resectable [2]. Neoadjuvant radiotherapy is more effective on well-oxygenated cells that cannot be surgically removed [3]. In approximately 25% of patients, postoperative adjuvant radiotherapy may be affected due to delayed postoperative recovery. However, delayed postoperative recovery does not affect the implementation of neoadjuvant radiotherapy [14, 4]. The use of neoadjuvant radiotherapy may help identify PDAC patients at high risk of early metastasis.

Therefore, neoadjuvant radiotherapy is considered to be applicable to borderline resectable PDAC and locally advanced PDAC. Some studies demonstrated that neoadjuvant radiotherapy can improve the R0 resection rate and the prognosis of patients with borderline resectable PDAC. After neoadjuvant radiotherapy, the median rate of resection and R0 resection of PDAC patients can reach 68 and 89% respectively. For patients who received neoadjuvant therapy and underwent surgical resection, the median OS range was 15.6 to 35 months. Compared with the group without neoadjuvant radiotherapy, the difference in median OS was statistically significant [15, 16]. In this study, patients with non-metastatic PDAC were divided into T1-3N0M0, T1-3N + M0, T4N0M0, T4N + M0 according to TNM stages, and the effects of different treatment regimens including neoadjuvant radiotherapy on the prognosis were analyzed. The results proved that neoadjuvant radiotherapy improves OS for T1-4N + M0/T4N0M0 PDAC patients. Moreover, for T4 patients, the effect of neoadjuvant radiotherapy on OS was significantly better than that of adjuvant radiotherapy and surgery plus chemotherapy. Therefore, the necessity of neoadjuvant radiotherapy should be emphasized in clinical practice for PDAC patients with stage T4.

However, the survival of T1-3N0M0 patients couldn’t benefit from neoadjuvant radiotherapy according to the results of this study. Some scholars also questioned the use of neoadjuvant radiotherapy in early PDAC. Patients with resectable PDAC can initially be surgically removed, but neoadjuvant radiotherapy may delay the patient’s surgical opportunity, making the lesions that could have been resected with R0 become unresectable or even distant metastases [17]. Especially in the process of neoadjuvant radiotherapy, if the patient has serious complications, such as biliary tract obstruction, this may aggravate the development of the disease, or even make the patient’s physical condition worse, not suitable for surgical treatment. Another problem that must be considered is that, unlike surgery, the initiation of neoadjuvant radiotherapy requires definite pathological results. Given the anatomical location and structure of the tumor, biopsy is sometimes difficult to perform and may delay treatment. The specificity of endoscopic ultrasound-guided biopsy is 96%, but the sensitivity is only 85.92% and repeated examinations are required in 11% of cases [18].

A prospective, randomized, controlled phase II trial in Germany comparing neoadjuvant chemoradiotherapy with surgical priority for resectable pancreatic cancer was prematurely discontinued after 73 patients were enrolled. The existing results showed that there was no significant difference in R0 removal rate and median overall survival time between the two groups [19]. A meta-analysis published in 2019 included 11 clinical studies involving 2666 patients from the university of Texas southwestern medical center, Montefiore medical center, erlangen university hospital, Germany, Tohoku university school of medicine, Japan, and others. The results showed that the R0 resection rate was improved in patients of resectable PDAC treated with neoadjuvant radiotherapy, but the overall survival time of the patients was not significantly increased [20]. Combined with the results of this study, the overall survival of surgery plus chemotherapy is significantly better than neoadjuvant radiotherapy and adjuvant radiotherapy, so it is recommended that patients with T1-3N0M0 should choose surgery plus chemotherapy as the priority.

A consensus has been reached on the mode of systemic therapy for PDAC under MDT [21]. For borderline resectable and locally advanced PDAC, neoadjuvant radiotherapy may transform patients who cannot be R0 resected or even inoperable into R0 resectable patients, thus extending survival time and benefiting the patients. In this study, a combination of neoadjuvant radiotherapy was recommended for patients with stage T4 PDAC. Whether neoadjuvant therapy can benefit patients with early resectable PDAC is still controversial. Our study suggested that T1-3N0M0 stage PDAC patients were preferred to receive surgery plus chemotherapy, while neoadjuvant radiotherapy was not recommended. In addition, T1-3N + M0 stage PDAC patients were preferentially recommended postoperative adjuvant radiotherapy. However, this study is only a retrospective analysis from a large database, and the results need to be further verified by prospective experiments. With the development of large clinical trials, high level of evidence-based medical evidence will continue to be presented, and the understanding of neoadjuvant radiotherapy for PDAC will be deepened, which may lead to a consensus on the existing controversies and treatment options in the future.

Similar to other studies using the SEER database as a data source, our study has limitations and requires careful interpretation of the results. First, while the SEER data included information about surgery, radiation, and chemotherapy, the details of these treatments (such as surgical margins, radiation dose, chemotherapy regiments, and chemotherapy sequence) were not recorded in the database. Second, the SEER database lacks some key clinical information that may be important for prognosis, such as tumor markers (CA19–9), the relationships between tumor and important blood vessels, and so on.

## Conclusions

In summary, this retrospective study analyzed SEER database cases from 2004 to 2016 and made the following recommendations: 1. Among patients with non-metastatic PDAC, stage T1-4N + M0/T4N0M0 patients who received neoadjuvant radiotherapy, adjuvant radiotherapy, and surgery plus chemotherapy had longer OS than those who received surgery alone, while stage T1-3N0M0 patients did not benefit from neoadjuvant radiotherapy. 2. For patients with stage T1-3N0M0, surgery plus chemotherapy is clinically recommended as the preferred treatment. 3. For PDAC patients with stage T1-3N + M0, postoperative adjuvant radiotherapy has a better prognosis and adjuvant radiotherapy is preferred. 4. For stage T4 patients, neoadjuvant radiotherapy had significantly longer OS than adjuvant radiotherapy and surgery plus chemotherapy, which may be appropriate for guidelines to adopt a more proactive stance on using of neoadjuvant radiotherapy for stage T4 PDAC patients.

## Supplementary information

**Additional file 1: Table 1.** Univariate and multivariate analyses of OS in the neoadjuvant radiotherapy group and the only surgery group for T1-3N0M0 PDAC patients.

**Additional file 2: Table 2.** Univariate and multivariate analyses of OS in the neoadjuvant radiotherapy group and the adjuvant radiotherapy group for T1-3N0M0 PDAC patients.

**Additional file 3: Table 3.** Univariate and multivariate analyses of OS in the neoadjuvant radiotherapy group and the surgery plus chemotherapy group for T1-3N0M0 PDAC patients.

**Additional file 4: Table 4.** Univariate and multivariate analyses of OS in the neoadjuvant radiotherapy group and the only surgery group for T1-3N + M0 PDAC patients.

**Additional file 5: Table 5.** Univariate and multivariate analyses of OS in the neoadjuvant radiotherapy group and the adjuvant radiotherapy group for T1-3N + M0 PDAC patients.

**Additional file 6: Table 6.** Univariate and multivariate analyses of OS in the neoadjuvant radiotherapy group and the surgery plus chemotherapy group for T1-3N + M0 PDAC patients.

**Additional file 7: Table 7.** Univariate and multivariate analyses of OS in the neoadjuvant radiotherapy group and the only surgery group for T4 PDAC patients.

**Additional file 8: Table 8.** Univariate and multivariate analyses of OS in the neoadjuvant radiotherapy group and the adjuvant radiotherapy group for T4 PDAC patients.

**Additional file 9: Table 9.** Univariate and multivariate analyses of OS in the neoadjuvant radiotherapy group and the surgery plus chemotherapy group for T4 PDAC patients.

## Data Availability

The data from this study is publicly available in the national cancer institute’s Surveillance, Epidemiology, and End Results (SEER) database at https://seer.cancer.gov/.

## Abbreviations

PDAC: Pancreatic Ductal Adenocarcinoma
OS: Overall Survival
SEER: Surveillance, Epidemiology, and End Results
CIs: Confidence intervals
HRs: Hazard ratios
MDT: Multi-disciplinary team
ISGPS: International Study Group on Pancreatic Surgery
US: United States
PSM: Propensity score matching
