# Supplementary material for: Effect of neoadjuvant radiotherapy on survival of non-metastatic pancreatic ductal adenocarcinoma: a SEER database analysis
Source: Radiat Oncol. 2020 May 13;15:107. doi: 10.1186/s13014-020-01561-z (PMC7222314; doi:10.1186/s13014-020-01561-z)
Supplement: Supplementary file 1 — Additional file 1: Table 1. Univariate and multivariate analyses of OS in the neoadjuvant radiotherapy group and the only surgery group for T1-3N0M0 PDAC patients. [file 13014_2020_1561_MOESM1_ESM.docx]

Table 1. Univariate and multivariate analyses of OS in the neoadjuvant radiotherapy group and the only surgery group for T1-3N0M0 PDAC patients.

|  |  | Before PSM | | | | After PSM | | | |
| --- | --- | --- | --- | --- | --- | --- | --- | --- | --- |
|  |  | Univariate analysis | Multivariate analysis | | | Univariate analysis | Multivariate analysis | | |
| Characteristics | Level | P | HR | 95%CI | P | P | HR | 95%CI | P |
| Insurance Recode | | <0.001 |  |  | <0.001 | 0.004 |  |  | 0.004 |
|  | Insured |  | Reference | Reference | Reference |  | Reference | Reference | Reference |
|  | No/unknown |  | 1.253 | 1.135-1.383 | <0.001 |  | 1.523 | 1.147-2.023 | 0.004 |
| Marital status |  | <0.001 |  |  | <0.001 | 0.624 |  |  | NA |
|  | Married |  | Reference | Reference | Reference |  |  |  |  |
|  | Single |  | 1.248 | 1.138-1.369 | <0.001 |  |  |  |  |
|  | Unknown |  | 1.017 | 0.820-1.261 | 0.880 |  |  |  |  |
| Age, years |  | <0.001 |  |  | <0.001 | <0.001 |  |  | <0.001 |
|  | <65 |  | Reference | Reference | Reference |  | Reference | Reference | Reference |
|  | ≥65 |  | 2.246 | 2.039-2.475 | <0.001 |  | 1.564 | 1.256-1.948 | <0.001 |
| Race recode |  | 0.004 |  |  | 0.784 | 0.819 |  |  | NA |
|  | White |  | Reference | Reference | Reference |  |  |  |  |
|  | Other |  | 1.016 | 0.908-1.136 | 0.784 |  |  |  |  |
| Sex |  | 0.023 |  |  | 0.001 | 0.356 |  |  | NA |
|  | Female |  | Reference | Reference | Reference |  |  |  |  |
|  | Male |  | 1.163 | 1.065-1.270 | 0.001 |  |  |  |  |
| Tumor site |  | <0.001 |  |  | <0.001 | 0.056 |  |  | NA |
|  | Pancreas Head | | Reference | Reference | Reference |  |  |  |  |
|  | Pancreas Body Tail | | 0.550 | 0.494-0.611 | <0.001 |  |  |  |  |
|  | Pancreas Other | | 0.725 | 0.635-0.828 | <0.001 |  |  |  |  |
| Grade |  | <0.001 |  |  | <0.001 | <0.001 |  |  | 0.001 |
|  | I |  | Reference | Reference | Reference |  | Reference | Reference | Reference |
|  | II |  | 2.950 | 2.597-3.352 | <0.001 |  | 1.549 | 1.002-2.394 | 0.049 |
|  | III/IV |  | 4.886 | 4.250-5.618 | <0.001 |  | 1.851 | 1.168-2.932 | 0.009 |
|  | Unknown |  | 1.654 | 1.413-1.936 | <0.001 |  | 1.054 | 0.672-1.653 | 0.819 |
| T stage |  | <0.001 |  |  | <0.001 | 0.192 |  |  | NA |
|  | T1 |  | Reference | Reference | Reference |  |  |  |  |
|  | T2 |  | 1.654 | 1.413-1.936 | <0.001 |  |  |  |  |
|  | T3 |  | 1.486 | 1.315-1.679 | <0.001 |  |  |  |  |
| Treatment methods | | <0.001 |  |  | 0.038 | 0.048 |  |  | 0.176 |
| Only surgery | |  | Reference | Reference | Reference |  | Reference | Reference | Reference |
| Neoadjuvant radiotherapy | | | 1.486 | 1.315-1.679 | 0.038 |  | 1.164 | 0.934-1.449 | 0.176 |
| Regional nodes examined | | <0.001 |  |  | 0.590 | 0.073 |  |  | NA |
|  | <15 |  | Reference | Reference | Reference |  |  |  |  |
|  | ≥15 |  | 0.956 | 0.866-1.055 | 0.367 |  |  |  |  |
|  | Unknown |  | 1.074 | 0.770-1.497 | 0.674 |  |  |  |  |
