# Supplementary material for: Effect of neoadjuvant radiotherapy on survival of non-metastatic pancreatic ductal adenocarcinoma: a SEER database analysis
Source: Radiat Oncol. 2020 May 13;15:107. doi: 10.1186/s13014-020-01561-z (PMC7222314; doi:10.1186/s13014-020-01561-z)
Supplement: Supplementary file 2 — Additional file 2: Table 2. Univariate and multivariate analyses of OS in the neoadjuvant radiotherapy group and the adjuvant radiotherapy group for T1-3N0M0 PDAC patients. [file 13014_2020_1561_MOESM2_ESM.docx]

Table 2. Univariate and multivariate analyses of OS in the neoadjuvant radiotherapy group and the adjuvant radiotherapy group for T1-3N0M0 PDAC patients.

|  |  | Before PSM | | | | After PSM | | | |
| --- | --- | --- | --- | --- | --- | --- | --- | --- | --- |
|  |  | Univariate analysis | Multivariate analysis | | | Univariate analysis | Multivariate analysis | | |
| Characteristics | Level | P | HR | 95%CI | P | P | HR | 95%CI | P |
| Insurance Recode | | 0.030 |  |  | 0.031 | 0.965 |  |  | NA |
|  | Insured |  | Reference | Reference | Reference |  |  |  |  |
|  | No/unknown |  | 1.153 | 1.013-1.311 | 0.031 |  |  |  |  |
| Marital status |  | 0.634 |  |  | NA | 0.832 |  |  | NA |
|  | Married |  |  |  |  |  |  |  |  |
|  | Single |  |  |  |  |  |  |  |  |
|  | Unknown |  |  |  |  |  |  |  |  |
| Age, years |  | <0.001 |  |  | 0.001 | 0.467 |  |  | NA |
|  | <65 |  | Reference | Reference | Reference |  |  |  |  |
|  | ≥65 |  | 1.219 | 1.086-1.369 | 0.001 |  |  |  |  |
| Race recode |  | 0.230 |  |  | NA | 0.850 |  |  | NA |
|  | White |  |  |  |  |  |  |  |  |
|  | Other |  |  |  |  |  |  |  |  |
| Sex |  | 0.028 |  |  | 0.057 | 0.687 |  |  | NA |
|  | Female |  | Reference | Reference | Reference |  |  |  |  |
|  | Male |  | 1.118 | 0.996-1.254 | 0.057 |  |  |  |  |
| Tumor site |  | 0.363 |  |  | NA | 0.592 |  |  | NA |
|  | Pancreas Head | |  |  |  |  |  |  |  |
|  | Pancreas Body Tail | |  |  |  |  |  |  |  |
|  | Pancreas Other | |  |  |  |  |  |  |  |
| Grade |  | 0.003 |  |  | <0.001 | 0.042 |  |  | 0.116 |
|  | I |  | Reference | Reference | Reference |  | Reference | Reference | Reference |
|  | II |  | 1.191 | 0.976-1.453 | 0.085 |  | 1.074 | 0.670-1.721 | 0.768 |
|  | III/IV |  | 1.191 | 0.976-1.453 | 0.002 |  | 1.365 | 0.831-2.244 | 0.219 |
|  | Unknown |  | 0.946 | 0.740-1.208 | 0.654 |  | 1.365 | 0.831-2.244 | 0.637 |
| T stage |  | 0.008 |  |  | 0.008 | 0.667 |  |  | NA |
|  | T1 |  | Reference | Reference | Reference |  |  |  |  |
|  | T2 |  | 1.165 | 0.994-1.365 | 0.059 |  |  |  |  |
|  | T3 |  | 1.331 | 1.110-1.595 | 0.002 |  |  |  |  |
| Treatment methods | | 0.016 |  |  | 0.006 | 0.039 |  |  | 0.125 |
| Neoadjuvant radiotherapy | |  | Reference | Reference | Reference |  | Reference | Reference | Reference |
| Adjuvant radiotherapy | | | 0.809 | 0.695-0.941 | 0.006 |  | 0.807 | 0.649-1.035 | 0.125 |
| Regional nodes examined | | 0.002 |  |  | 0.004 | 0.919 |  |  | NA |
|  | <15 |  | Reference | Reference | Reference |  |  |  |  |
|  | ≥15 |  | 0.816 | 0.718-0.927 | 0.002 |  |  |  |  |
|  | Unknown |  | 1.229 | 0.793-1.904 | 0.356 |  |  |  |  |
