# Supplementary material for: Effect of neoadjuvant radiotherapy on survival of non-metastatic pancreatic ductal adenocarcinoma: a SEER database analysis
Source: Radiat Oncol. 2020 May 13;15:107. doi: 10.1186/s13014-020-01561-z (PMC7222314; doi:10.1186/s13014-020-01561-z)
Supplement: Supplementary file 3 — Additional file 3: Table 3. Univariate and multivariate analyses of OS in the neoadjuvant radiotherapy group and the surgery plus chemotherapy group for T1-3N0M0 PDAC patients. [file 13014_2020_1561_MOESM3_ESM.docx]

Table 3. Univariate and multivariate analyses of OS in the neoadjuvant radiotherapy group and the surgery plus chemotherapy group for T1-3N0M0 PDAC patients.

|  |  | Before PSM | | | | After PSM | | | |
| --- | --- | --- | --- | --- | --- | --- | --- | --- | --- |
|  |  | Univariate analysis | Multivariate analysis | | | Univariate analysis | Multivariate analysis | | |
| Characteristics | Level | P | HR | 95%CI | P | P | HR | 95%CI | P |
| Insurance Recode | | 0.001 |  |  | 0.012 | 0.813 |  |  | NA |
|  | Insured |  | Reference | Reference | Reference |  |  |  |  |
|  | No/unknown |  | 1.210 | 1.043-1.404 | 0.012 |  |  |  |  |
| Marital status |  | 0.637 |  |  | NA | 0.817 |  |  | NA |
|  | Married |  |  |  |  |  |  |  |  |
|  | Single |  |  |  |  |  |  |  |  |
|  | Unknown |  |  |  |  |  |  |  |  |
| Age, years |  | <0.001 |  |  | <0.001 | 0.001 |  |  | 0.002 |
|  | <65 |  | Reference | Reference | Reference |  | Reference | Reference | Reference |
|  | ≥65 |  | 1.298 | 1.160-1.453 | <0.001 |  | 1.422 | 1.133-1.785 | 0.002 |
| Race recode |  | 0.745 |  |  | NA | 0.559 |  |  | NA |
|  | White |  |  |  |  |  |  |  |  |
|  | Other |  |  |  |  |  |  |  |  |
| Sex |  | 0.985 |  |  | NA | 0.255 |  |  | NA |
|  | Female |  |  |  |  |  |  |  |  |
|  | Male |  |  |  |  |  |  |  |  |
| Tumor site |  | 0.274 |  |  | NA | 0.583 |  |  | NA |
|  | Pancreas Head | |  |  |  |  |  |  |  |
|  | Pancreas Body Tail | |  |  |  |  |  |  |  |
|  | Pancreas Other | |  |  |  |  |  |  |  |
| Grade |  | <0.001 |  |  | <0.001 | 0.002 |  |  | 0.003 |
|  | I |  | Reference | Reference | Reference |  | Reference | Reference | Reference |
|  | II |  | 1.549 | 1.266-1.896 | <0.001 |  | 1.451 | 0.936-2.250 | 0.096 |
|  | III/IV |  | 2.135 | 1.736-2.627 | <0.001 |  | 1.451 | 0.936-2.250 | 0.005 |
|  | Unknown |  | 1.282 | 1.009-1.630 | 0.042 |  | 1.152 | 0.734-1.808 | 0.539 |
| T stage |  | <0.001 |  |  | <0.001 | 0.157 |  |  | NA |
|  | T1 |  | Reference | Reference | Reference |  |  |  |  |
|  | T2 |  | 1.282 | 1.009-1.630 | <0.001 |  |  |  |  |
|  | T3 |  | 1.647 | 1.390-1.952 | <0.001 |  |  |  |  |
| Treatment methods | | 0.001 |  |  | 0.001 | 0.034 |  |  | 0.025 |
| Surgery plus chemotherapy | |  | Reference | Reference | Reference |  | Reference | Reference | Reference |
| Neoadjuvant radiotherapy | | | 1.285 | 1.107-1.491 | 0.001 |  | 1.280 | 1.045-1.574 | 0.025 |
| Regional nodes examined | | 0.007 |  |  | 0.037 | 0.019 |  |  | 0.043 |
|  | <15 |  | Reference | Reference | Reference |  | Reference | Reference | Reference |
|  | ≥15 |  | 0.885 | 0.507-0.991 | 0.011 |  | 0.935 | 0.722-1.210 | 0.609 |
|  | Unknown |  | 0.866 | 0.488-1.535 | 0.621 |  | 2.521 | 1.649-3.070 | 0.015 |
