# Supplementary material for: Effect of neoadjuvant radiotherapy on survival of non-metastatic pancreatic ductal adenocarcinoma: a SEER database analysis
Source: Radiat Oncol. 2020 May 13;15:107. doi: 10.1186/s13014-020-01561-z (PMC7222314; doi:10.1186/s13014-020-01561-z)
Supplement: Supplementary file 4 — Additional file 4: Table 4. Univariate and multivariate analyses of OS in the neoadjuvant radiotherapy group and the only surgery group for T1-3N + M0 PDAC patients. [file 13014_2020_1561_MOESM4_ESM.docx]

Table 4. Univariate and multivariate analyses of OS in the neoadjuvant radiotherapy group and the only surgery group for T1-3N+M0 PDAC patients.

|  |  | Before PSM | | | | After PSM | | | |
| --- | --- | --- | --- | --- | --- | --- | --- | --- | --- |
|  |  | Univariate analysis | Multivariate analysis | | | Univariate analysis | Multivariate analysis | | |
| Characteristics | Level | P | HR | 95%CI | P | P | HR | 95%CI | P |
| Insurance Recode | | <0.001 |  |  | 0.004 | 0.977 |  |  | NA |
|  | Insured |  | Reference | Reference | Reference |  |  |  |  |
|  | No/unknown |  | 1.140 | 1.044-1.245 | 0.004 |  |  |  |  |
| Marital status |  | <0.001 |  |  | 0.016 | 0.414 |  |  | NA |
|  | Married |  | Reference | Reference | Reference |  |  |  |  |
|  | Single |  | 1.122 | 1.038-1.213 | 0.004 |  |  |  |  |
|  | Unknown |  | 1.063 | 0.864-1.309 | 0.562 |  |  |  |  |
| Age, years |  | <0.001 |  |  | <0.001 | 0.001 |  |  | <0.001 |
|  | <65 |  | Reference | Reference | Reference |  | Reference | Reference | Reference |
|  | ≥65 |  | 1.504 | 1.382-1.637 | <0.001 |  | 1.612 | 1.235-2.104 | <0.001 |
| Race recode |  | 0.100 |  |  | 0.784 | 0.600 |  |  | NA |
|  | White |  | Reference | Reference | Reference |  |  |  |  |
|  | Other |  | 1.016 | 0.908-1.136 | 0.784 |  |  |  |  |
| Sex |  | 0.697 |  |  | 0.001 | 0.434 |  |  | NA |
|  | Female |  | Reference | Reference | Reference |  |  |  |  |
|  | Male |  | 1.163 | 1.065-1.270 | 0.001 |  |  |  |  |
| Tumor site |  | <0.001 |  |  | <0.001 | 0.211 |  |  | NA |
|  | Pancreas Head | | Reference | Reference | Reference |  |  |  |  |
|  | Pancreas Body Tail | | 0.650 | 0.578-0.730 | <0.001 |  |  |  |  |
|  | Pancreas Other | | 0.843 | 0.741-0.958 | 0.009 |  |  |  |  |
| Grade |  | <0.001 |  |  | <0.001 | <0.001 |  |  | <0.001 |
|  | I |  | Reference | Reference | Reference |  | Reference | Reference | Reference |
|  | II |  | 3.138 | 2.726-3.612 | <0.001 |  | 2.337 | 1.335-4.090 | 0.003 |
|  | III/IV |  | 4.180 | 3.618-4.828 | <0.001 |  | 3.270 | 1.846-5.793 | <0.001 |
|  | Unknown |  | 1.855 | 1.515-2.271 | <0.001 |  | 1.573 | 0.818-3.022 | 0.174 |
| T stage |  | <0.001 |  |  | <0.001 | 0.199 |  |  | NA |
|  | T1 |  | Reference | Reference | Reference |  |  |  |  |
|  | T2 |  | 1.300 | 1.150-1.470 | <0.001 |  |  |  |  |
|  | T3 |  | 1.442 | 1.260-1.650 | <0.001 |  |  |  |  |
| N stage |  | <0.001 |  |  | <0.001 | 0.412 |  |  | NA |
|  | N1 |  | Reference | Reference | Reference |  |  |  |  |
|  | N2 |  | 1.226 | 1.132-1.329 | <0.001 |  |  |  |  |
| Treatment methods | | 0.002 |  |  | 0.008 | 0.049 |  |  | 0.036 |
| Only surgery | |  | Reference | Reference | Reference |  | Reference | Reference | Reference |
| Neoadjuvant radiotherapy | | | 0.795 | 0.671-0.942 | 0.008 |  | 0.618 | 0.429-0.863 | 0.036 |
| Regional nodes examined | | 0.915 |  |  | NA | 0.399 |  |  | NA |
|  | <15 |  |  |  |  |  |  |  |  |
|  | ≥15 |  |  |  |  |  |  |  |  |
|  | Unknown |  |  |  |  |  |  |  |  |
