# Supplementary material for: Effect of neoadjuvant radiotherapy on survival of non-metastatic pancreatic ductal adenocarcinoma: a SEER database analysis
Source: Radiat Oncol. 2020 May 13;15:107. doi: 10.1186/s13014-020-01561-z (PMC7222314; doi:10.1186/s13014-020-01561-z)
Supplement: Supplementary file 5 — Additional file 5: Table 5. Univariate and multivariate analyses of OS in the neoadjuvant radiotherapy group and the adjuvant radiotherapy group for T1-3N + M0 PDAC patients. [file 13014_2020_1561_MOESM5_ESM.docx]

Table 5. Univariate and multivariate analyses of OS in the neoadjuvant radiotherapy group and the adjuvant radiotherapy group for T1-3N+M0 PDAC patients.

|  |  | Before PSM | | | | After PSM | | | |
| --- | --- | --- | --- | --- | --- | --- | --- | --- | --- |
|  |  | Univariate analysis | Multivariate analysis | | | Univariate analysis | Multivariate analysis | | |
| Characteristics | Level | P | HR | 95%CI | P | P | HR | 95%CI | P |
| Insurance Recode | | 0.006 |  |  | 0.009 | 0.165 |  |  | NA |
|  | Insured |  | Reference | Reference | Reference |  |  |  |  |
|  | No/unknown |  | 1.125 | 1.030-1.229 | 0.009 |  |  |  |  |
| Marital status |  | 0.765 |  |  | NA | 0.980 |  |  | NA |
|  | Married |  |  |  |  |  |  |  |  |
|  | Single |  |  |  |  |  |  |  |  |
|  | Unknown |  |  |  |  |  |  |  |  |
| Age, years |  | 0.101 |  |  | NA | 0.570 |  |  | NA |
|  | <65 |  |  |  |  |  |  |  |  |
|  | ≥65 |  |  |  |  |  |  |  |  |
| Race recode |  | 0.704 |  |  | NA | 0.067 |  |  | NA |
|  | White |  |  |  |  |  |  |  |  |
|  | Other |  |  |  |  |  |  |  |  |
| Sex |  | <0.001 |  |  | 0.001 | 0.318 |  |  | NA |
|  | Female |  | Reference | Reference | Reference |  |  |  |  |
|  | Male |  | 1.139 | 1.054-1.231 | 0.001 |  |  |  |  |
| Tumor site |  | 0.936 |  |  | NA | 0.114 |  |  | NA |
|  | Pancreas Head | |  |  |  |  |  |  |  |
|  | Pancreas Body Tail | |  |  |  |  |  |  |  |
|  | Pancreas Other | |  |  |  |  |  |  |  |
| Grade |  | <0.001 |  |  | <0.001 | 0.247 |  |  | NA |
|  | I |  | Reference | Reference | Reference |  |  |  |  |
|  | II |  | 1.439 | 1.239-1.670 | <0.001 |  |  |  |  |
|  | III/IV |  | 1.733 | 1.489-2.018 | <0.001 |  |  |  |  |
|  | Unknown |  | 1.234 | 0.988-1.540 | 0.064 |  |  |  |  |
| T stage |  | <0.001 |  |  | <0.001 | 0.724 |  |  | NA |
|  | T1 |  | Reference | Reference | Reference |  |  |  |  |
|  | T2 |  | 1.321 | 1.169-1.493 | <0.001 |  |  |  |  |
|  | T3 |  | 1.472 | 1.288-1.682 | <0.001 |  |  |  |  |
| N stage |  | <0.001 |  |  | <0.001 | 0.004 |  |  | 0.002 |
|  | N1 |  | Reference | Reference | Reference |  | Reference | Reference | Reference |
|  | N2 |  | 1.472 | 1.288-1.682 | <0.001 |  | 1.709 | 1.212-2.411 | 0.002 |
| Treatment methods | | 0.019 |  |  | 0.015 | 0.040 |  |  | 0.022 |
| Adjuvant radiotherapy | |  | Reference | Reference | Reference |  | Reference | Reference | Reference |
| Neoadjuvant radiotherapy | | | 1.047 | 1.013-1.706 | 0.015 |  | 1.364 | 1.046-1.777 | 0.022 |
| Regional nodes examined | | <0.001 |  |  | <0.001 | 0.340 |  |  | NA |
|  | <15 |  | Reference | Reference | Reference |  |  |  |  |
|  | ≥15 |  | 0.772 | 0.712-0.836 | <0.001 |  |  |  |  |
|  | Unknown |  | 0.928 | 0.586-1.469 | 0.749 |  |  |  |  |
