# Supplementary material for: Effect of neoadjuvant radiotherapy on survival of non-metastatic pancreatic ductal adenocarcinoma: a SEER database analysis
Source: Radiat Oncol. 2020 May 13;15:107. doi: 10.1186/s13014-020-01561-z (PMC7222314; doi:10.1186/s13014-020-01561-z)
Supplement: Supplementary file 6 — Additional file 6: Table 6. Univariate and multivariate analyses of OS in the neoadjuvant radiotherapy group and the surgery plus chemotherapy group for T1-3N + M0 PDAC patients. [file 13014_2020_1561_MOESM6_ESM.docx]

Table 6. Univariate and multivariate analyses of OS in the neoadjuvant radiotherapy group and the surgery plus chemotherapy group for T1-3N+M0 PDAC patients.

|  |  | Before PSM | | | | After PSM | | | |
| --- | --- | --- | --- | --- | --- | --- | --- | --- | --- |
|  |  | Univariate analysis | Multivariate analysis | | | Univariate analysis | Multivariate analysis | | |
| Characteristics | Level | P | HR | 95%CI | P | P | HR | 95%CI | P |
| Insurance Recode | | 0.042 |  |  | 0.121 | 0.260 |  |  | NA |
|  | Insured |  | Reference | Reference | Reference |  |  |  |  |
|  | No/unknown |  | 1.086 | 0.978-1.206 | 0.121 |  |  |  |  |
| Marital status |  | 0.680 |  |  | NA | 0.300 |  |  | NA |
|  | Married |  |  |  |  |  |  |  |  |
|  | Single |  |  |  |  |  |  |  |  |
|  | Unknown |  |  |  |  |  |  |  |  |
| Age, years |  | <0.001 |  |  | <0.001 | 0.025 |  |  | 0.007 |
|  | <65 |  | Reference | Reference | Reference |  | Reference | Reference | Reference |
|  | ≥65 |  | 1.168 | 1.084-1.260 | <0.001 |  | 1.430 | 1.102-1.854 | 0.007 |
| Race recode |  | 0.525 |  |  | NA | 0.595 |  |  | NA |
|  | White |  |  |  |  |  |  |  |  |
|  | Other |  |  |  |  |  |  |  |  |
| Sex |  | 0.522 |  |  | NA | 0.940 |  |  | NA |
|  | Female |  |  |  |  |  |  |  |  |
|  | Male |  |  |  |  |  |  |  |  |
| Tumor site |  | 0.405 |  |  | NA | 0.930 |  |  | NA |
|  | Pancreas Head | |  |  |  |  |  |  |  |
|  | Pancreas Body Tail | |  |  |  |  |  |  |  |
|  | Pancreas Other | |  |  |  |  |  |  |  |
| Grade |  | <0.001 |  |  | <0.001 | 0.011 |  |  | <0.001 |
|  | I |  | Reference | Reference | Reference |  | Reference | Reference | Reference |
|  | II |  | 1.279 | 1.103-1.483 | 0.001 |  | 2.359 | 1.329-4.185 | 0.003 |
|  | III/IV |  | 1.672 | 1.441-1.940 | <0.001 |  | 3.410 | 1.907-6.100 | 0.000 |
|  | Unknown |  | 1.176 | 0.959-1.443 | 0.119 |  | 2.506 | 1.342-4.679 | 0.004 |
| T stage |  | <0.001 |  |  | <0.001 | 0.656 |  |  | NA |
|  | T1 |  | Reference | Reference | Reference |  |  |  |  |
|  | T2 |  | 1.305 | 1.157-1.471 | <0.001 |  |  |  |  |
|  | T3 |  | 1.390 | 1.218-1.586 | <0.001 |  |  |  |  |
| N stage |  | <0.001 |  |  | <0.001 | 0.204 |  |  | NA |
|  | N1 |  | Reference | Reference | Reference |  |  |  |  |
|  | N2 |  | 1.325 | 1.225-1.434 | <0.001 |  |  |  |  |
| Treatment methods | | 0.035 |  |  | 0.795 | 0.021 |  |  | 0.541 |
| Surgery plus chemotherapy | |  | Reference | Reference | Reference |  | Reference | Reference | Reference |
| Neoadjuvant radiotherapy | | | 1.023 | 0.863-1.212 | 0.795 |  | 1.083 | 0.838-1.400 | 0.541 |
| Regional nodes examined | | <0.001 |  |  | <0.001 | 0.314 |  |  | NA |
|  | <15 |  | Reference | Reference | Reference |  |  |  |  |
|  | ≥15 |  | 0.826 | 0.765-0.892 | <0.001 |  |  |  |  |
|  | Unknown |  | 0.761 | 0.470-1.233 | 0.267 |  |  |  |  |
