# Supplementary material for: Effect of neoadjuvant radiotherapy on survival of non-metastatic pancreatic ductal adenocarcinoma: a SEER database analysis
Source: Radiat Oncol. 2020 May 13;15:107. doi: 10.1186/s13014-020-01561-z (PMC7222314; doi:10.1186/s13014-020-01561-z)
Supplement: Supplementary file 7 — Additional file 7: Table 7. Univariate and multivariate analyses of OS in the neoadjuvant radiotherapy group and the only surgery group for T4 PDAC patients. [file 13014_2020_1561_MOESM7_ESM.docx]

Table 7. Univariate and multivariate analyses of OS in the neoadjuvant radiotherapy group and the only surgery group for T4 PDAC patients.

|  |  | Before PSM | | | | After PSM | | | |
| --- | --- | --- | --- | --- | --- | --- | --- | --- | --- |
|  |  | Univariate analysis | Multivariate analysis | | | Univariate analysis | Multivariate analysis | | |
| Characteristics | Level | P | HR | 95%CI | P | P | HR | 95%CI | P |
| Insurance Recode | | 0.017 |  |  | 0.275 | 0.529 |  |  | NA |
|  | Insured |  | Reference | Reference | Reference |  |  |  |  |
|  | No/unknown |  | 1.161 | 0.888-1.518 | 0.275 |  |  |  |  |
| Marital status |  | 0.011 |  |  | 0.624 | 0.149 |  |  | NA |
|  | Married |  | Reference | Reference | Reference |  |  |  |  |
|  | Single |  | 1.007 | 0.780-1.302 | 0.955 |  |  |  |  |
|  | Unknown |  | 1.294 | 0.767-2.181 | 0.334 |  |  |  |  |
| Age, years |  | <0.001 |  |  | <0.001 | 0.134 |  |  | NA |
|  | <65 |  | Reference | Reference | Reference |  |  |  |  |
|  | ≥65 |  | 1.752 | 1.378-2.228 | <0.001 |  |  |  |  |
| Race recode |  | 0.999 |  |  | NA | 0.470 |  |  | NA |
|  | White |  |  |  |  |  |  |  |  |
|  | Other |  |  |  |  |  |  |  |  |
| Sex |  | 0.629 |  |  | NA | 0.662 |  |  | NA |
|  | Female |  |  |  |  |  |  |  |  |
|  | Male |  |  |  |  |  |  |  |  |
| Tumor site |  | 0.723 |  |  | NA | 0.628 |  |  | NA |
|  | Pancreas Head | |  |  |  |  |  |  |  |
|  | Pancreas Body Tail | |  |  |  |  |  |  |  |
|  | Pancreas Other | |  |  |  |  |  |  |  |
| Grade |  | 0.709 |  |  | NA | 0.107 |  |  | NA |
|  | I |  |  |  |  |  |  |  |  |
|  | II |  |  |  |  |  |  |  |  |
|  | III/IV |  |  |  |  |  |  |  |  |
|  | Unknown |  |  |  |  |  |  |  | NA |
| N stage |  | <0.001 |  |  | 0.002 | 0.121 |  |  |  |
|  | N0 |  | Reference | Reference | Reference |  |  |  |  |
|  | N1 |  | 1.485 | 1.146-1.925 | 0.003 |  |  |  |  |
|  | N2 |  | 1.780 | 1.219-2.597 | 0.003 |  |  |  |  |
| Treatment methods | | <0.001 |  |  | <0.001 | <0.001 |  |  | <0.001 |
| Only surgery | |  | Reference | Reference | Reference |  | Reference | Reference | Reference |
| Neoadjuvant radiotherapy | | | 0.459 | 0.349-0.602 | <0.001 |  | 0.466 | 0.331-0.657 | <0.001 |
| Regional nodes examined | | 0.409 |  |  | NA | 0.729 |  |  | NA |
|  | <15 |  |  |  |  |  |  |  |  |
|  | ≥15 |  |  |  |  |  |  |  |  |
|  | Unknown |  |  |  |  |  |  |  |  |
