# Supplementary material for: Effect of neoadjuvant radiotherapy on survival of non-metastatic pancreatic ductal adenocarcinoma: a SEER database analysis
Source: Radiat Oncol. 2020 May 13;15:107. doi: 10.1186/s13014-020-01561-z (PMC7222314; doi:10.1186/s13014-020-01561-z)
Supplement: Supplementary file 8 — Additional file 8: Table 8. Univariate and multivariate analyses of OS in the neoadjuvant radiotherapy group and the adjuvant radiotherapy group for T4 PDAC patients. [file 13014_2020_1561_MOESM8_ESM.docx]

Table 8. Univariate and multivariate analyses of OS in the neoadjuvant radiotherapy group and the adjuvant radiotherapy group for T4 PDAC patients.

|  |  | Before PSM | | | | After PSM | | | |  |
| --- | --- | --- | --- | --- | --- | --- | --- | --- | --- | --- |
|  |  | Univariate analysis | Multivariate analysis | | | Univariate analysis | Multivariate analysis | | |  |
| Characteristics | Level | P | HR | 95%CI | P | P | HR | 95%CI | P |  |
| Insurance Recode | | 0.001 |  |  | 0.004 | 0.052 |  |  | NA |  |
|  | Insured |  | Reference | Reference | Reference |  |  |  |  |  |
|  | No/unknown |  | 1.468 | 1.133-1.902 | 0.004 |  |  |  |  |  |
| Marital status |  | 0.768 |  |  | NA | 0.731 |  |  | NA |  |
|  | Married |  |  |  |  |  |  |  |  |  |
|  | Single |  |  |  |  |  |  |  |  |  |
|  | Unknown |  |  |  |  |  |  |  |  |  |
| Age, years |  | 0.439 |  |  | NA | 0.866 |  |  | NA |  |
|  | <65 |  |  |  |  |  |  |  |  |  |
|  | ≥65 |  |  |  |  |  |  |  |  |  |
| Race recode |  | 0.459 |  |  | NA | 0.728 |  |  | NA |  |
|  | White |  |  |  |  |  |  |  |  |  |
|  | Other |  |  |  |  |  |  |  |  |  |
| Sex |  | 0.041 |  |  | 0.042 | 0.302 |  |  | NA |  |
|  | Female |  | Reference | Reference | Reference |  |  |  |  |  |
|  | Male |  | 1.277 | 1.009-1.615 | 0.042 |  |  |  |  |  |
| Tumor site |  | 0.733 |  |  | NA | 0.473 |  |  | NA |  |
|  | Pancreas Head | |  |  |  |  |  |  |  |  |
|  | Pancreas Body Tail | |  |  |  |  |  |  |  |  |
|  | Pancreas Other | |  |  |  |  |  |  |  |  |
| Grade |  | 0.136 |  |  | NA | 0.005 |  |  | <0.001 |  |
|  | I |  |  |  |  |  | Reference | Reference | Reference | |
|  | II |  |  |  |  |  | 1.991 | 0.709-5.595 | 0.191 |  |
|  | III/IV |  |  |  |  |  | 4.269 | 1.547-11.785 | 0.005 |  |
|  | Unknown |  |  |  |  |  | 3.031 | 1.067-8.612 | 0.037 |  |
| N stage |  | 0.013 |  |  | 0.615 | 0.492 |  |  | NA |  |
|  | N0 |  | Reference | Reference | Reference |  |  |  |  |  |
|  | N1 |  | 0.936 | 0.709-1.234 | 0.637 |  |  |  |  |  |
|  | N2 |  | 1.104 | 0.786-1.551 | 0.568 |  |  |  |  |  |
| Treatment methods | | <0.001 |  |  | <0.001 | 0.002 |  |  | 0.002 |  |
| Adjuvant radiotherapy | |  | Reference | Reference | Reference |  | Reference | Reference | Reference | |
| Neoadjuvant radiotherapy | | | 0.590 | 0.445-0.781 | <0.001 |  | 0.589 | 0.419-0.830 | 0.002 |  |
| Regional nodes examined | | 0.085 |  |  | NA | 0.733 |  |  | NA |  |
|  | <15 |  |  |  |  |  |  |  |  |  |
|  | ≥15 |  |  |  |  |  |  |  |  |  |
|  | Unknown |  |  |  |  |  |  |  |  |  |
