# Supplementary material for: Effect of neoadjuvant radiotherapy on survival of non-metastatic pancreatic ductal adenocarcinoma: a SEER database analysis
Source: Radiat Oncol. 2020 May 13;15:107. doi: 10.1186/s13014-020-01561-z (PMC7222314; doi:10.1186/s13014-020-01561-z)
Supplement: Supplementary file 9 — Additional file 9: Table 9. Univariate and multivariate analyses of OS in the neoadjuvant radiotherapy group and the surgery plus chemotherapy group for T4 PDAC patients. [file 13014_2020_1561_MOESM9_ESM.docx]

Table 9. Univariate and multivariate analyses of OS in the neoadjuvant radiotherapy group and the surgery plus chemotherapy group for T4 PDAC patients.

|  |  | Before PSM | | | | After PSM | | | |
| --- | --- | --- | --- | --- | --- | --- | --- | --- | --- |
|  |  | Univariate analysis | Multivariate analysis | | | Univariate analysis | Multivariate analysis | | |
| Characteristics | Level | P | HR | 95%CI | P | P | HR | 95%CI | P |
| Insurance Recode | | 0.101 |  |  | NA | 0.532 |  |  | NA |
|  | Insured |  |  |  |  |  |  |  |  |
|  | No/unknown |  |  |  |  |  |  |  |  |
| Marital status |  | 0.005 |  |  | 0.080 | 0.674 |  |  | NA |
|  | Married |  | Reference | Reference | Reference |  |  |  |  |
|  | Single |  | 1.257 | 0.971-1.627 | 0.083 |  |  |  |  |
|  | Unknown |  | 1.731 | 0.884-3.392 | 0.110 |  |  |  |  |
| Age, years |  | 0.189 |  |  | NA | 0.818 |  |  | NA |
|  | <65 |  |  |  |  |  |  |  |  |
|  | ≥65 |  |  |  |  |  |  |  |  |
| Race recode |  | 0.832 |  |  | NA | 0.717 |  |  | NA |
|  | White |  |  |  |  |  |  |  |  |
|  | Other |  |  |  |  |  |  |  |  |
| Sex |  | 0.328 |  |  | NA | 0.216 |  |  | NA |
|  | Female |  |  |  |  |  |  |  |  |
|  | Male |  |  |  |  |  |  |  |  |
| Tumor site |  | 0.236 |  |  | NA | 0.996 |  |  | NA |
|  | Pancreas Head | |  |  |  |  |  |  |  |
|  | Pancreas Body Tail | |  |  |  |  |  |  |  |
|  | Pancreas Other | |  |  |  |  |  |  |  |
| Grade |  | 0.799 |  |  | NA | 0.677 |  |  | NA |
|  | I |  |  |  |  |  |  |  |  |
|  | II |  |  |  |  |  |  |  |  |
|  | III/IV |  |  |  |  |  |  |  |  |
|  | Unknown |  |  |  |  |  |  |  |  |
| N stage |  | <0.001 |  |  | <0.001 | 0.119 |  |  | NA |
|  | N0 |  | Reference | Reference | Reference |  |  |  |  |
|  | N1 |  | 1.412 | 1.076-1.852 | 0.013 |  |  |  |  |
|  | N2 |  | 2.464 | 1.736-3.497 | <0.001 |  |  |  |  |
| Treatment methods | | <0.001 |  |  | 0.040 | 0.028 |  |  | 0.028 |
| Surgery plus chemotherapy | |  | Reference | Reference | Reference |  | Reference | Reference | Reference |
| Neoadjuvant radiotherapy | | | 0.752 | 0.573-0.987 | 0.040 |  | 0.707 | 0.519-0.963 | 0.028 |
| Regional nodes examined | | <0.025 |  |  | 0.001 | 0.468 |  |  | NA |
|  | <15 |  | Reference | Reference | Reference |  |  |  |  |
|  | ≥15 |  | 0.641 | 0.499-0.822 | <0.001 |  |  |  |  |
|  | Unknown |  | 1.131 | 0.601-2.130 | 0.703 |  |  |  |  |
